# Global Gene Expression Analysis of the Zoonotic Parasite *Trichinella spiralis* Revealed Novel Genes in Host Parasite Interaction

**DOI:** 10.1371/journal.pntd.0001794

**Published:** 2012-08-28

**Authors:** Xiaolei Liu, Yanxia Song, Ning Jiang, Jielin Wang, Bin Tang, Huijun Lu, Shuai Peng, Zhiguang Chang, Yizhi Tang, Jigang Yin, Mingyuan Liu, Yan Tan, Qijun Chen

**Affiliations:** 1 Key Laboratory of Zoonosis, Ministry of Education, Institute of Zoonosis, Jilin University, Changchun, People's Republic of China; 2 Central Laboratory, The First Affiliated Hospital, Jilin University, Changchun, People's Republic of China; 3 Laboratory of Parasitology, Institute of Pathogen Biology, Chinese Academy of Medical Sciences/Peking Union Medical College, Beijing, People's Republic of China; University of South Florida, United States of America

## Abstract

**Background:**

Trichinellosis is a typical food-borne zoonotic disease which is epidemic worldwide and the nematode *Trichinella spiralis* is the main pathogen. The life cycle of *T. spiralis* contains three developmental stages, i.e. adult worms, new borne larva (new borne L1 larva) and muscular larva (infective L1 larva). Stage-specific gene expression in the parasites has been investigated with various immunological and cDNA cloning approaches, whereas the genome-wide transcriptome and expression features of the parasite have been largely unknown. The availability of the genome sequence information of *T. spiralis* has made it possible to deeply dissect parasite biology in association with global gene expression and pathogenesis.

**Methodology and Principal Findings:**

In this study, we analyzed the global gene expression patterns in the three developmental stages of *T. spiralis* using digital gene expression (DGE) analysis. Almost 15 million sequence tags were generated with the Illumina RNA-seq technology, producing expression data for more than 9,000 genes, covering 65% of the genome. The transcriptome analysis revealed thousands of differentially expressed genes within the genome, and importantly, a panel of genes encoding functional proteins associated with parasite invasion and immuno-modulation were identified. More than 45% of the genes were found to be transcribed from both strands, indicating the importance of RNA-mediated gene regulation in the development of the parasite. Further, based on gene ontological analysis, over 3000 genes were functionally categorized and biological pathways in the three life cycle stage were elucidated.

**Conclusions and Significance:**

The global transcriptome of *T. spiralis* in three developmental stages has been profiled, and most gene activity in the genome was found to be developmentally regulated. Many metabolic and biological pathways have been revealed. The findings of the differential expression of several protein families facilitate understanding of the molecular mechanisms of parasite biology and the pathological aspects of trichinellosis.

## Author Summary

Trichinellosis of human and other mammals was caused through the ingestion of the parasite *Trichinella sparilis* in contaminated meat. It is a typical zoonotic disease that affects more than 10 million people world-wide. Parasites of the genus *Trichinella* are unique intracellular pathogens. Adult *Trichinella* parasites directly release newborn larvae which invade striated muscle cells and causes diseases. In this study, we profiled the global transcriptome in the three developmental stages of *T. spiralis*. The transcriptomic analysis revealed the global gene expression patterns from newborn larval stage through muscle larval stage to adults. Thousands of genes with stage-specific transcriptional patterns were described and novel genes involving host-parasite interaction were identified. More than 45% of the protein-coding genes showed evidence of transcription from both sense and antisense strands which suggests the importance of RNA-mediated gene regulation in the parasite. This study presents a first deep analysis of the transcriptome of *T. spiralis*, providing insight information of the parasite biology.

## Introduction

*Thichinella spiralis* is often referred as one of the largest intracellular parasite that cause trichinellosis with an estimation of more than 10 million people infected world-wide [1], [2]. Like many other food-borne zoonotic parasites, *T. spiralis* exists in several life-cycle stages. The complex life cycle of *T. spiralis* is completed in two niches, the intra-multicellular niche in intestinal epithelium (adults, Ad) and the intracellular niche in the skeletal muscle fibers (muscle larvae, ML). After being ingested with infected muscle tissue, the ML are released and revived in the small intestine, which invade the epithelial layer where they mature, mate and produce the newborn larvae (NBL). The NBL migrate through the lymphatic and blood vessels, invade striated muscle cells and develop into the ML, which is infective to the next host [3]. Thus, unlike other nematodes, *T. spiralis* are ovoviviparous [4], [5], which makes them evolutionary divergent from other nematodes previously analyzed by genomic approaches [6], especially the well-characterized free-living worm *Caenorhabditis elegans*. *C. elegans* is the first multicellular organism, of which the genome has been sequenced and phylogenetically classified in Clade V [7]. *T. spiralis* is a member of clade I that diverged early in the evolution of the *Nematoda*, with remarkably different biological and molecular characteristics from other nematodes [6].

Previous analysis of gene expression of *T. spiralis* at different developmental stages has mainly been carried out by sequencing of expression sequence tag (EST) [6]. However, the fragmentary data of ESTs are insufficient for a full understanding of the parasite biology. Recent study indicated that *T. spiralis* has a much smaller genome with an estimation of 64 Mb in nucleic DNA, coding for around 15,808 proteins [8]. The availability of the genomic information has made it possible for a deep dissection of the parasite's basic biology. In recent years, next-generation sequencing (NGS) techniques have dramatically improved the efficiency and the speed of gene discovery [9], [10]. NGS technology generates millions of short sequence reads from a single instrumental run which can be effectively assembled and employed for gene discovery and comparison of gene expression patterns. Further, NGS allows for a detection of genes with very low expression levels. It has been frequently used to characterize specific gene families or genetic pathways.

In this study, we compared gene expression variations among the three developmental stages of *T. spiralis* using next generation sequencing technology. Thousands of genes, especially the genes involving in host/parasite interactions and parasite development were identified. The data will facilitate discovery of potential vaccine and drug targets of *T. spiralis*.

## Materials and Methods

### Parasites and RNA purification

Muscle larvae (ML) of *T. spiralis* (strain ISS534) were obtained from rats at 35 days post infection by digestion of minced skeletal muscle according to the previously described method [11], [12]. The adult worms and newborn larvae were collected as described previously [12], [13]. The study of using laboratory animals was reviewed and approved by the Ethical Committee of Jilin University affiliated to the Provincial Animal Health Committee, Jilin Province, China (Ethical Clearance number IZ-2009-008). All animal work was conducted according to the guidelines of the Chinese Law of Animal Protection (Section 6).

Total RNA of *T. spiralis* (Ad, NBL and ML) was purified using Trizol reagent (Invitrogen, CA, USA) according to the manufacturer's instructions. RNAs were dissolved in Diethylpyrocarbonate (DEPC)-treated water and treated with DNase I (Invitrogen, CA, USA). Total RNA was quantified by measuring the absorbance at 260 nm with a Nanodrop 1000 machine (Thermo Scientific CA, USA).

### Digital gene expression (DGE) library construction and sequencing

The DGE libraries were prepared by using the Illumina gene expression sample preparation kit [14]. Briefly, 6 µg of total RNA from each preparation was treated with Oligo-(dT) conjugated magnetic beads to purify mRNA. Double-strand cDNA was synthesized guided by the Oligo-(dT) as a primer and digested with the endonuclease Nla*III* that recognizes the CATG sites. The Illumina adaptor 1, containing a Mme*I* restriction site, was added to the cDNAs attached to the magnetic beads, which was further digested with Mme*I*. Following Mme*I* digestion and dephosphorylation, cDNA fragments were purified and the Illumina adaptor 2 was ligated to the 3′ends of the tags to create tag library with different adapters at both ends. After15 cycles of linear PCR amplification, the 95 bp fragments were purified from 6% TBE PAGE gels and attached to the Illumina sequencing chip for sequencing.

### Analysis and mapping of DGE tags

After removal of low quality and adaptor sequences, the clean 21 bp tag sequences containing CATG were mapped onto the reference genome sequences, allowing for no more than one nucleotide mismatch. To compare the differences in gene expression in three DGE libraries, the tag frequency was statistically analyzed according to the method FDR (False Discovery Rate), a method used to determine the threshold of P-value in multiple tests [15]. A FDR<0.001 and an absolute value of the log2Radio>1 was used as the threshold to judge significant differences in gene expression. KEGG Ontology (KO) of the transcripts was identified trough blasting the KEGG database. Gene Ontology (GO) analysis of all differentially expressed genes was performed by searching in the GO database. The enriched p-values of KO and GO were calculated according to the hypergeometric test [16]:
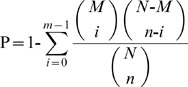
In this equation, *N* means the number of genes with GO/KO annotation, *n* means the number of differentially expressed genes in *N*, *M* means the number of genes in each GO/KO term, and *m* represents the number of differentially expressed gene in each GO/KO term. For GO enrichment analysis, all P-values were treated with Bonferroni correction. We selected a corrected p-value,0.05 as a threshold to determine significant enrichment of the gene sets. In contrast, for KO enrichment analysis, we used a FDR,0.05 as a threshold to determine significant enrichment of the gene sets. WEGO was employed to make a GO classification [17].

Phylogenetic tree was built for DNase II protein families using PHYLIP (version 3.69; [18]) after aligning the family members with CLUSTAL X (version 2.1). And a neighbor joining tree was generated using PHYLIP-NEIGHBOR. Then, the phylogenetic tree was visualized and edited using the Tree Figure Drawing Tool *- FigTree* (version 1.3.1).

### Verification of stage-specific transcripts by quantitative real-time PCR

Total RNA of *T. spiralis* (muscle larvae, adult worms and newborn larvae) was extracted using Trizol reagent (Invitrogen, CA, USA) and treated with Dnase I (Invitrogen, CA, USA). The RNAs were dissolved in Diethylpyrocarbonate (DEPC)-treated water and reverse transcribed with 200 U SuperScript™ III Reverse Transcriptase (Invitrogen) according to the manufacturer's instructions. The specific primers were listed in File S1 as forward and reverse primers based on the stage-specifically expressed genes. The GAPDH gene was used as an endogenous reference. The qPCR was performed using the SYBR Kit (Applied Biosystems, Foster City, CA, USA) according to the manufacturer's protocol using an Applied Biosystems 7500 detection system. The relative expression was analyzed using the SDS1.4 software (Applied Biosystems, Foster City, USA).

## Results

### Library construction and sequencing

To generate digital gene expression signatures of *T. spiralis* at different developmental stages, DGE libraries were generated from the three developmental stages of the parasite, and sequenced using Solexa (Illumina) high through-put technology. A total of 5,289,863 tags from muscular larvae (ML), 5,214,135 tags from adult worms (Ad), 5,055,659 tags from newborn larvae (NBL) were obtained (Table 1). After filtering the low quality tags and adaptor sequences, the total number of clean tags in ML, Ad and NBL were 5,077,645 (96.0% of total tags), 5,003,105 (96.0% of total tags), 4,849,883 (96.0% of total tags), respectively (Table 1). The sequence data has been submitted to the GEO website (ftp://ftp-private.ncbi.nlm.nih.gov/fasp/) with an accession number of GSE39151.

**Table 1:** Expression profiles of sequence tags in three differential stages.

Summary	Ad	ML	NBL
Total Tag	5,214,135	5,289,863	5,055,659
Clean Tag	5,003,105	5,077,645	4,849,883
Tag mapped to gene	2,670,150	2,187,559	2,254,426
Identified genes	8,131	8,554	8,881
Redundancy (%)*	98.4	98.4	97.9

* The redundancy of the three libraries was calculated according to the formula (Redundancy = 100−(Total Clean Distinct Tags/Total Tags×100).

Heterogeneity and redundancy are two significant characteristics of gene expression in the three developmental stages of *T. spiralis*, which have previously been observed in other metazoans [19]. The distribution of clean tags in the three libraries shows a consistent pattern, with most of the tags coming from highly expressed genes. The percentage of distinct tags with high counts dropped dramatically and the distinct tags with more than 100 copies accounted less than 10%. However, more than 75% of total clean tags have an account above 100 (**File S2**).

The clean tags were then mapped onto the draft genome of *T. spiralis* (ftp://ftp.ncbi.nlm.nih.gov/genbank/wgs/wgs.ABIR.1.gbff.gz) [8] and the numbers of tags that could be mapped onto genes with no more than one base pair mismatch in Ad, ML, NBL were 2,670,150, 2,187,559 and 2,254,426, respectively (Table 1). In total, around 10,000 genes were identified from the three libraries, accounted for approximately 65% of genes in the annotated genome [8] (File S3).

### Differentially expressed genes in three developmental stages of *T. spiralis*

**Figure 1:**
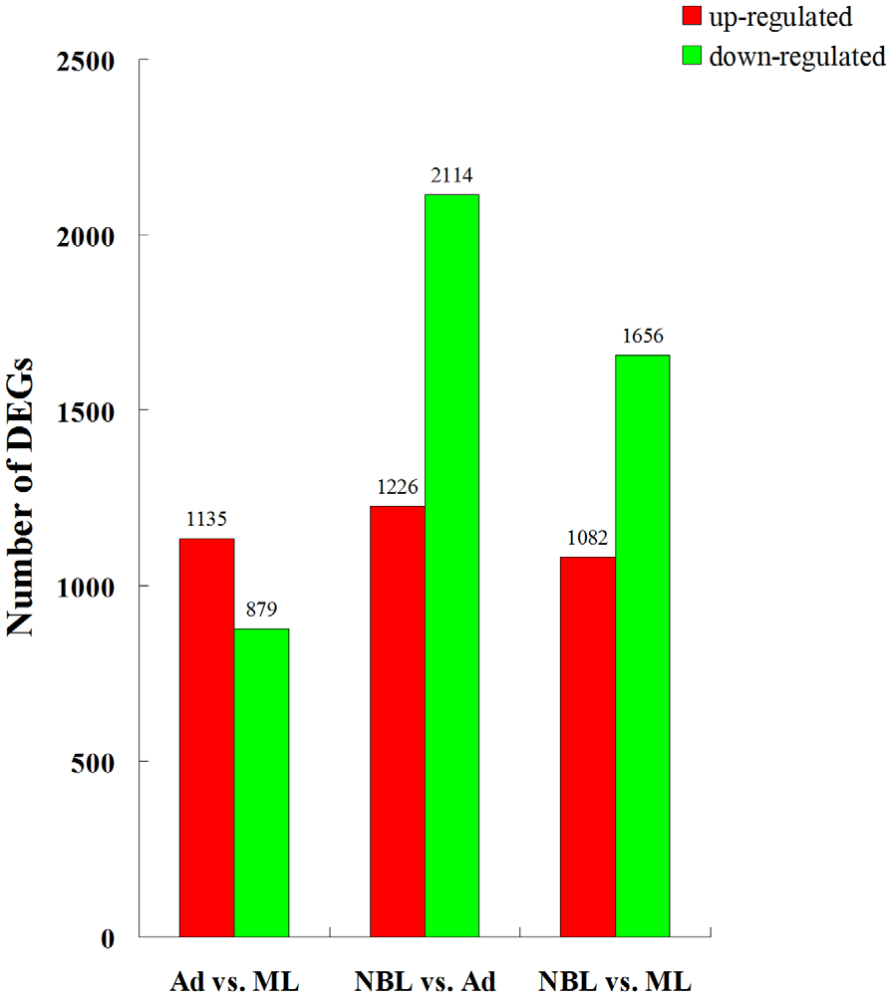
Numbers of differentially expressed genes in each comparison. The numbers of up-regulated (in red) and down-regulated genes (in green) are presented. The number of genes up-regulated in the NBL stage is significantly more than that of the other two stages.

**Figure 2:**
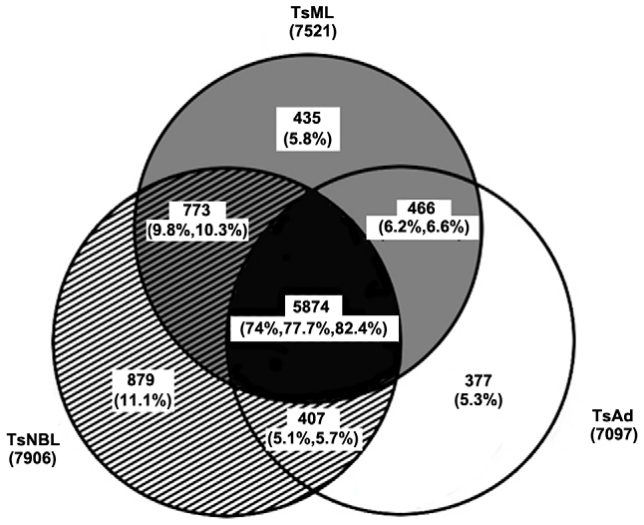
Distribution of genes commonly and stage-specifically expressed in the three developmental stages. Gene numbers and percentages (in brackets) identified in Ad, ML and NBL are presented.

To identify genes that differentially expressed in the three developmental stages, gene expression variations were analyzed by pair-wise (Ad versus ML, NBL versus Ad, and NBL versus ML) comparison of the sequence tags. A number of genes were found differentially expressed between the developmental stages (Figure 1 and File S4). The number of differentially expressed genes between NBL and Ad was more than that between Ad and ML. And the number of up-regulated genes expressed in NBL was more than that in the other two stages. Apart from the differentially expressed genes, a number of stage-specific genes were identified and the number of stage-specific genes expressed in NBL was twice as much as that of Ad or ML (Figure 2).

**Figure 3:**
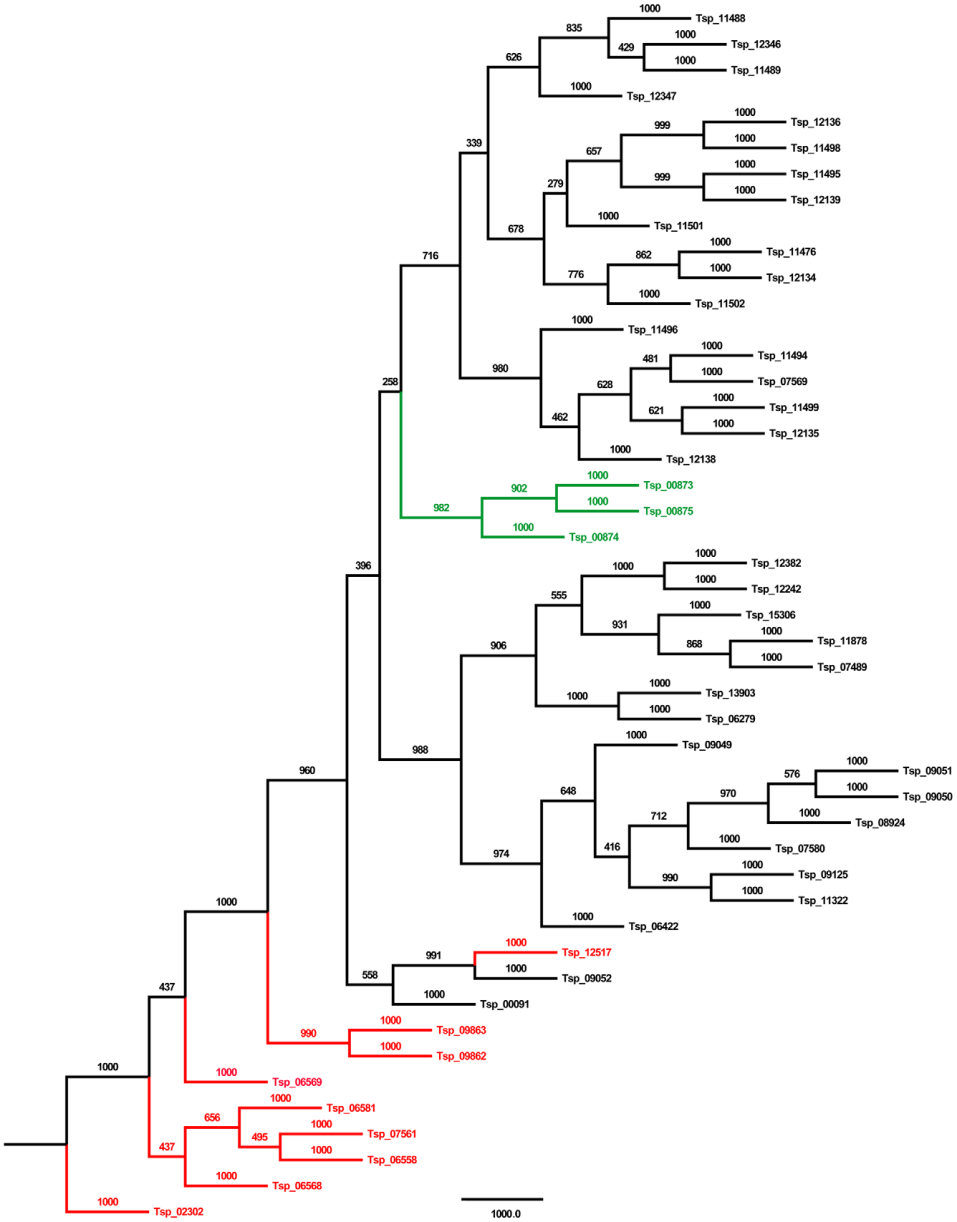
Phylogenetic analysis of DNase II family proteins identified in *T. spiralis*. Amino acid sequences of 47 DNase II were aligned by using CLASTAL X (version 2.1) and phylogenetic tree was constructed using PHYLIP (version 3.6.9). Homologous proteins with stage-specific expression were highlighted in color (Ad-specific DNase II homologues in red, ML-specific DNase II homologues in blue, and NBL-specific homologues in black). Scale bar indicates amino acid substitutions in the sequences and evolutionary distance.

**Figure 4:**
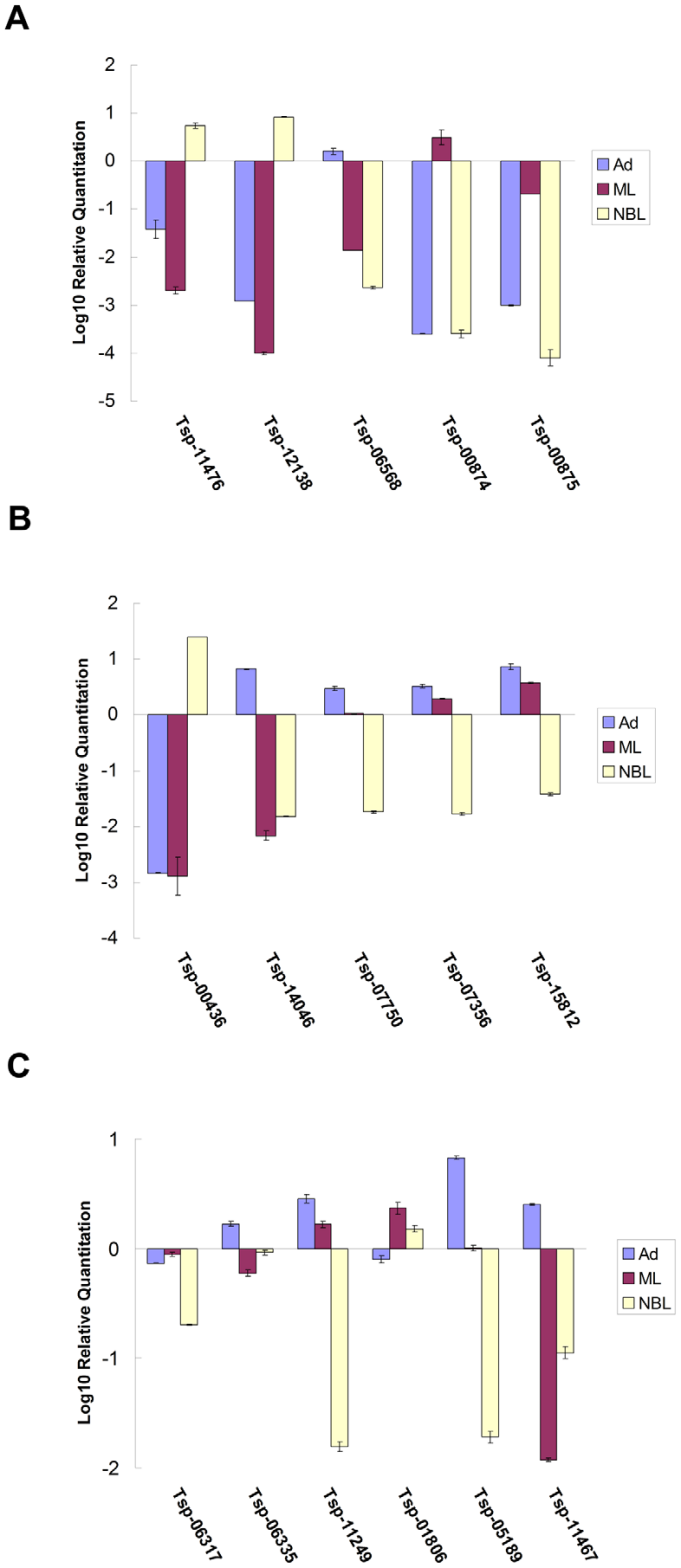
Confirmation of the differentially expressed genes in the three stages of *T. spiralis by Real-time PCR.* The expression of 5 genes encoding serine proteases (A), 5 genes encoding DNase II (B) and 6 genes encoding heat shock protein A (Tsp_06317), MIF (Tsp_06335), cystatin (Tsp_11249), systeine-glycine (Tsp_01806) and two unknown proteins (Tsp_05189 and Tsp_11467) (C) in Ad (light blue), ML (purple) and NBL (orange) was analyzed by quantitative real-time PCR. Numbers of mRNA transcripts relative to the standard were with log10 scale in plus (up-regulation and minus (down-regulation).

Among the differentially expressed genes, genes coding for families of proteases such as astacin protease, serine protease, and DNase II (Table 2, and File S4 and S5) were more prominent. Serine protease and DNase II constitute the two excretory-secretory (E-S) protein families **involved** in host-parasite interactions in trichinellosis [6], [20]. The two gene families showed obviously stage-specific variations in expression in three developmental stages. 47 differentially expressed DNase II family genes were identified and most of these genes were up-regulated in NBL compared to the other stages (Figure 3, Table 2 and File S6). In contrast, only 6 DNase II genes were up-regulate in ML compared to the other stages (Table 2 and File S6). Further, most of these genes have homology with 27 previously identified DNase II homologues (File S6) [20]. Tsp_11476 and Tsp_12138 showed very high expression in NBL, while Tsp_06568 was detected in rather high abundance in Ad; and Tsp_00874 and Tsp_00875 were mainly expressed in ML (Figure 4A). Another interesting gene family is that encode serine protease. Contrast to DNase II family, most of serine protease genes were up-regulated in Ad compared to the other stages. In the three developmental stages of the parasite, a large number of serine protease family genes showed stage-specific expression, especially Tsp_00436, Tsp_15812, Tsp_07356, Tsp_07750 and Tsp_14046 (Figure 4B and Table 2).

**Table 2:** Numbers of genes differentially expressed in the three developmental stages of *T. spiralis*.

Gene family/Metabolic pathways	Up-regulation			Down-regulation		
	Ad	ML	NBL	Ad	ML	NBL
DNase II	12	6	31	35	41	15
Serine proteases	13	13	10	17	13	18
Serpins	6	3	15	12	13	3
Zinc metalloproteinases	12	6	31	35	41	15
SOD	1	1	2	3	2	2
Glycolysis	1	0	3	3	4	0
Anaerobic metabolism	0	0	2	2	2	0
Citric acid cycle	5	5	0	1	1	7

Apart from the DNase II and serine protease families, genes encoding zinc metalloprotease, serine protease inhibitor (serpin), the heat shock protein (HSP), macrophage migration inhibitory factor (MIF) and antioxidant enzymes were also identified. Zinc metalloprotease is high homologous with nematode astacin protease. Genes coding for serpin and zinc metalloprotease showed a similar stage-specific expression pattern with up-regulation in NBL rather than the other stages, especially the genes like tsp_00173, tsp_01570, tsp_06688, tsp_09479, tsp_04481, tsp_01304, tsp_00804 and tsp_03942. Superoxide dismutase (SOD) and glutathione perxidase are two important antioxidant enzymes which protect the parasite from reactive oxygen species. The genes encoding these enzymes also showed stage-specific expression. The genes tsp_01933 (SOD) and tsp_06126 (SOD) showed very high expression in NBL, while tsp_11103 (SOD) and tsp_02268 (glutathione perxidase) were detected in rather high abundance in ML. Tsp_06335 encoding MIF and tsp_11249 encoding cystatin were mainly expressed in Ad. The gene encoding HSP 70 (Tsp_06317) was found up-regulated in ML (Table 2 and File S7).

The enzymes involved in metabolisms showed obviously stage-specific in transcription pattern. Phosphofructokinase (tsp_05639), enolase (tsp_09466) and Pyruvate Kinase (tsp_08030) were up-regulated in NBL. Whereas, the expression of Tsp_08363 encoding the major hexokinase isoenzyme showed no significant differences. Tsp_05267 encoded the minor hexokinase isoenzyme and was mainly expressed in Ad and ML. Lactate dehydrogenase (tsp_08060) and phosphoenolpyruvate carboxykinase (tsp_007989), the key enzymes in anaerobic metabolism, were up-regulated in NBL rather than the other stages. Whereas Two genes (tsp_03114, tsp_08643) encoding subunits of pyruvate dehydrogenase, which associated with glycolysis in the citric acid cycle via conversion of pyruvate to acetyl-CoA were mainly expressed in Ad and ML. Citrate synthase (tsp_01728) and isocitrate dehydrogenase (tsp_05617) were up-regulated in Ad. Another isocitrate dehydrogenase (tsp_06181) was up-regulated in ML (Table 2 and File S7).

### Confirmation of gene expression by real-time PCR

In order to verify the genes that were actually differentially expressed in the three developmental stages, the expression of 16 genes respectively coding for DNase II (Tsp_11476, Tsp_12138, Tsp_06568, Tsp_00874 and Tsp_00875), serine protease (Tsp_00436, Tsp_15812, Tsp_07356, Tsp_07750 and Tsp_14046), and 6 genes respectively encoding heat shock protein A (Tsp_06317), macrophage migration inhibitory factor (MIF) (Tsp_06335), cystatin (Tsp_11249), systeine-glycine (Tsp_01806) and two genes with unknown functions (Tsp_05189 and Tsp_11467) were analyzed by quantitative real-time PCR. The q-PCR results confirmed the data obtained in the sequencing analysis (Figure 4 A, B and C).

### Functional annotation of differentially expressed genes

**Figure 5:**
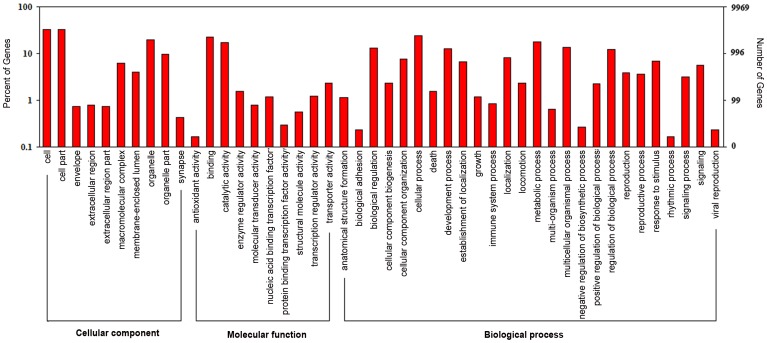
GO categories of the genes identified. Y-axis (left) represents percentages of genes identified in this study; and Y-axis (right) represents the actual gene number. The genes were annotated in three main categories: biological process, cellular component and molecular function (X-axis).

Among the transcripts of the 9,969 genes identified, 35% (3,496) could be assigned into one or more GO categories which were consistent with previous studies [8]. The remaining uncharacterized genes are likely to fulfill novel functions. In each of the three main categories (molecular function, cellular component and biological process) of the GO classification, ‘binding and catalytic activity’, ‘cell and cell part’ and ‘cellular process and metabolic process’ are dominant (Figure 5 and File S8).

In KO classification, 156 differentially expressed genes were significantly enriched in Ad versus ML, 408 genes in NBL versus ML and 395 genes in NBL versus Ad. Most of these genes encode proteins participating in ‘metabolism’, ‘environmental information processing’, ‘cellular processes’ and ‘organism systems’, i.e. glycolysis/gluconeogenesis, oocyst meiosis, calcium signaling pathway, vascular smooth muscle, insulin signaling pathway and lysosome (File S9). The comparison between Ad and the other two stages revealed that most of the expression of genes related to oocyst meiosis was up-regulated in Ad, while most of the genes correlated to glycolysis/gluconeogenesis, calcium signaling pathway and vascular smooth muscle contraction were up-regulated in NBL instead of Ad and ML.

### Antisense transcripts of *T. spiralis*

One of the advantages of DGE technique is that it could reveal transcripts with strand specificity [14]. We thus analyzed the sense and antisense transcripts of the transcriptome obtained. Of the 9,969 genes identified in this study, approximately 70% showed evidence of transcription in both orientations. The antisense transcripts obtained in Ad, ML and NBL were 6,303, 6,690, 7,033, respectively (File S10). While the transcripts from both strands obtained in Ad, ML and NBL were 5,269, 5,657 and 6,058, respectively. Among these genes, 2,682, 2,818 and 3,105 genes had tags corresponding to sense strands more than that from antisense strands in Ad, ML and NBL. Further, 2,476, 2,662 and 2,867 genes had more tags from antisense strands than that from sense strands in Ad ML and NBL stages (Table 3 and File S10).

**Table 3:** Numbers of genes with transcripts from both strands.

	Transcripts from both strands	More transcripts from sense strands	More transcripts from sense strands
Ad	5,269	2,682	2,476
ML	5,657	2,818	2,662
NBL	6,508	3,105	2,867

## Discussion

The availability of the draft genomic sequence of *T. spiralis* has made it possible to deeply investigate the global gene expression and gene regulation mechanisms in the development of the parasite. By comparing the DGE libraries obtained from three developmental stages of *T. spiralis*, we have identified a large number of functionally important genes with differential expression features.

First, like in other organisms with multiple developmental stages, the genome of *T. spiralis* is developmentally regulated. More than 70% of the genes in the genome were found activated in all three developmental stages, of which between 5–11% of genes are preferentially expressed at different stages. In general, more transcripts were identified in the new borne larvae, suggesting that genes at this stage are more active (Figure 1, 2, Table 1–2 and File S2). Unlike adult worms, the new-borne larvae will need to invade intestine epithelium and establish new niches in the tissue, thus the parasite need a different biological arsenal from the adult stage for adaptation and development in the host.

Secondly, functional analysis of the transcripts revealed a large number of genes encoding excretory-secretory proteins, especially families of parasite-derived DNase II and proteases. Previous studies have indicated that families of serine proteases and DNase II of *T. spiralis* were important parasitic components in host/parasite interactions and modulation of host immune responses [20]–[24]. Serine proteases have been proved to be critical for invasion of the mammalian host cells by *trypanosoma cruzi* and *steinernema carpocapsae* [25]–[28]. Here, we found 30 genes coding for homologous serine proteases were differentially expressed in the development of the parasite (Table 2 and Files S4 and S5). With the identification of the stage-specific expression of these proteases, it is now possible to further explore their importance in parasitization and as target for intervention such as vaccine development.

DNase II belongs to the acidic endonuclease family. They are essential nucleases nucleases that exist in non-metazoan, fulfilling a variety of functions from digesting DNA of apoptotic cell corpses and dietary DNA to modulating host immune responses [24]. They possess a histidine-rich domain which is believed to be the functional core of the protein family. Though there is more than one copy of DNase II genes in many organisms, the number of DNase II genes in *T. spiralis* is an exception with 47 copies identified in the strain studied. Phylogenetic analysis showed that these DNases exist in different subgroups (Figure 3). In light of the findings of their expression variation in different developmental stages, it is postulated that different variants fulfill different functionality. Recent studies suggested **that** DNase II in *T. spiralis* may function as self-protective molecules through modulation of host innate immune responses by cleaving DNA from apoptotic host cells [20], [29]. Deep investigation of functional specialty of the DNase II family members will be able to reveal mechanisms of host-parasite interaction and pathogenesis of trichinellosis.

Apart from the above protease and DNase II families, genes encoding heat shock protein 70 (HSP), macrophage migration inhibitory factor (MIF), systeine-glycine protein and cystatin were also found to be expressed in stage-specific manner. These molecules have been previously reported to be important in host/parasite interactions [30]–[35]. It has been proved that the parasites produce HSPs upon heat or oxidative stress that are related to resistance to harsh environment changes and are therefore beneficial to their survival [30]. MIF is a cytokine ubiquitous in mammals. However, many pathogens have genes encoding MIF homologues and the function of pathogen-derived MIF is believed to modulate host immune responses [35]. Thus it is not a surprise that *T. spiralis* also possesses this gene. Cystatins is a kind of cysteine protease inhibitor and has been reported as an important immuno-modulatory factor that contributes to the immune evasion strategies of the parasites [31].

Zinc-dependent metalloprotease of *T. spiralis* showed significant homology to the astacin metalloprotease family of *Caenorhabditis elegan*. Astacin metalloprotease have diverse functions including hydrolysis of extracellular matrix components such as type I collagen [36]. Since the parasites need to degrade the fibrinogen and plasminogen when they invade the epithelial and muscul cells (Ad and ML) or migrate to the small mesenteric veins (NBL), zinc-dependent metalloproteinases might be one of the effecter molecules in the process of tissue invasion. Serpins are a large protein family and can inactivate proteinases by forming complexes with serine proteinase. It has been reported that serpins could inhibit the host immune response and protects many parasites to evade the host immune defense [37]–[39]. Among the developmental stages, NBL is most fragile but is more exposed to the host immune system. That may explain why the genes encoding the zinc-dependent metalloprotease and serpin were up-regulated in NBL. Theses enzymes can help NBL for a more efficient penetration of host defensive barriers and, in the meantime, avoid the immune attack.

SOD and glutathione perxidase are two main important antioxidants. SOD can catalyze the conversion of superoxide anion into hydrogen peroxide and molecular oxygen, while glutathione perxidases catalyze the reduction of hydrogen peroxide along with oxidation of glutathione to glutathione disulfide. Secretion of antioxidant enzymes is believed to protect the parasite from reactive oxygen species which arise from host phagocytes [40]. Like DNase II and serine protease families, a number of genes encoding SOD and glutathione perxidases were identified and some of them showed different expression pattern in various developmental stages of *T. spiralis*. It is suggested that the DNase II, serine protease, SOD and glutathione perxidase were all contributed to self-protection of the parasite during invasion.

Thirdly, functional annotation of differentially expressed genes has partially revealed biological processes in different developmental stages of the parasite (Figure 5 and File S9). For example, oocyst meiosis is directly involved in the reproduction of the parasites. Therefore, most of differentially expressed genes related to oocyst meiosis were up-regulated in Ad compared to the other two stages, which was likely due to the preparation for the production of eggs. On the other hand, glycolysis/gluconeogenesis, calcium signaling pathway and vascular smooth muscle contraction pathways are mainly involved in energy metabolism, development and structure of tissue regeneration. Thus genes associated with these pathways were found up-regulated in NBL instead of Ad and ML due to the rapid growth.

Previous study indicated that there is a shift from anaerobic to aerobic metabolism in the developmental stage from ML to Ad [41]–[43], whereas no related information about the metabolism of NBL is available. In this study, we analyzed the differential expression pattern of the genes encoding the key enzymes involved in energy metabolisms. Phosphofructokinase, enolase and Pyruvate Kinase are essential enzymes in glycolysis. Genes coding for the three enzymes were up-regulated in NBL which indicated that energy metabolism were more activated in the NBL stage. Lactate dehydrogenase is a critical enzyme in anaerobic metabolism which can catalyze the conversion of pyruvate into lactate. Another key enzyme in anaerobic metabolism is phosphoenolpyruvate carboxykinase which can catalyze the conversion of phosphoenolpyruvate (PEP) into oxaloacetate and the reverse citric acid cycle. The genes encoding the two enzymes were found mainly expressed in NBL. Thus it is likely that T. spiralis adapted mainly an anaerobic metabolism in NBL stage. On the other hand, the expression of genes encoding pyruvate dehydrogenase, citrate synthase and isocitrate dehydrogenase, critical enzymes involved in citric acid cycle in aerobic metabolism, were up-regulated in Ad. No obvious significant differences in the expression of genes involved in the citric acid cycle and anaerobic metabolism between ML and Ad was observed.

Lastly, recent studies have revealed widespread expression of complementary sense-antisense transcript pairs by transcriptome sequencing [14], [44]–[47] and antisense-mediated gene regulation in developmental processes of different organisms have been proposed [48]–[50]. More than 7000 of the protein-coding genes were found bi-directionally transcribed in *T. spiralis*, accounted for approximately 70% of the identified genes in this study and 45% of all estimated genes in the genome, which was similar to that observed in S. *japonicum*, mice and human [14], [44], [45], [51]. To our knowledge, this is the first study to observe such abundant antisense transcripts in *T. spiralis*. Since the antisense transcripts were also polyadenylated, antisense RNAs can encode proteins [45], [51], [52]. However, most antisense transcriptions in the mammalian genome were found to be non-protein-coding RNAs [45], [49], [51]. Though the function of antisense transcripts has not been well understood, functional validation studies indicated that antisense transcripts were **a** heteromorphous group with common features and may modulate the expression level of the sense transcripts or influence the sense mRNA processing [53]. The mechanism of such regulation remains unknown; however, a mechanism of gene regulation by natural antisense transcripts (NAT) derived endogenous siRNAs (endo-siRNAs) has recently emerged. Endogenous siRNAs derived from transposable elements, NAT and long hairpin RNAs have been identified in *Drosophila* and mouse and *S. japonicum*. Endo-siRNAs can silence homologous transcripts by RNA interference (RNAi). Therefore, it is likely that natural antisense transcripts as sources of endo-siRNAs possess similar regulatory function in *T. spiralis* as in other organisms [13].

In summary, approximately 65% of genes in *T. spiralis* genome were identified and a large number of functionally interesting genes were discovered and analyzed in the three developmental stages of the parasite through high through-put RNA sequencing techniques. More than 45% of the protein-coding genes showed evidence of transcription from both sense and antisense strands. The data from this study has paved a way for deep investigation of the parasite biology and host parasite interaction.

## Supporting Information

File S1 **Primers used in qRT-PCR for validation of differentially expressed genes.**
(XLS)

File S2 **Distribution of total clean tags and distinct clean tags in each DGE library.** The numbers in square brackets indicate the range of copy numbers for each category of tags. The data in parentheses indicate the number and percentage of corresponding tags among the total or distinct clean tags. Distribution of total clean tags and distinct clean tags in NBL, ML and Ad stages are presented.
(TIF)

File S3 **List of all genes identified by DGE.** The first column represents names of the identified genes. *TS-Ad_raw*, *TS-ML_raw* and *TS-NBL_raw* mean the number of tags detected by sequencing in the three developmental stages, respectively. *TS-Ad_norm*, *TS-Ad_norm* and *TS-Ad norm* mean total times of detected tags per million in three developmental stages, respectively. GO Component, GO Function and GO Process mean the three main categories (cellular component, molecular function and biological process) in the GO classification, respectively.
(XLS)

File S4 **List of differentially expressed genes in the three developmental stages.**
(XLS)

File S5 **The top ten differentially expressed genes in each library of comparisons.**
(XLS)

File S6 **The DNase II family genes differentially expressed in each library.**
(XLS)

File S7 **Genes encoding proteinases and proteins in metabolic pathways.**
(XLS)

File S8 **GO annotation of genes.** The first column represents the names of the main GO categories. The second column lists the names of the secondary GO-terms. The last column represents the numbers and percentages of genes annotated to the given GO-terms.
(XLS)

File S9 **KO annotation of genes.** The first column represents the pathways and classified functional annotations. The second column represents the number of differentially expressed genes belonging to the pathway. The third column represents the number of all genes belonging to the pathway. ‘Genes’ means the gene involved in the annotated pathways. ‘KOs’ means the KEGG Ontology belonging to the pathway.
(XLS)

File S10 **Tags mapped to either sense (+), antisense strand (−) or both strands of the genes identified.** ‘GeneExpression (+)’ and ‘GeneExpression (−)’ mean the expression level of sense and antisense genes, respectively. ‘TPM (+)’ and ‘TPM (−)’ mean the total times of detected tags per million of the sense and antisense strands.
(XLS)
