# Supplementary figures and images for: Global Gene Expression Analysis of the Zoonotic Parasite Trichinella spiralis Revealed Novel Genes in Host Parasite Interaction
Source: PLoS Negl Trop Dis. 2012 Aug 28;6(8):e1794. doi: 10.1371/journal.pntd.0001794 (PMC3429391; doi:10.1371/journal.pntd.0001794)

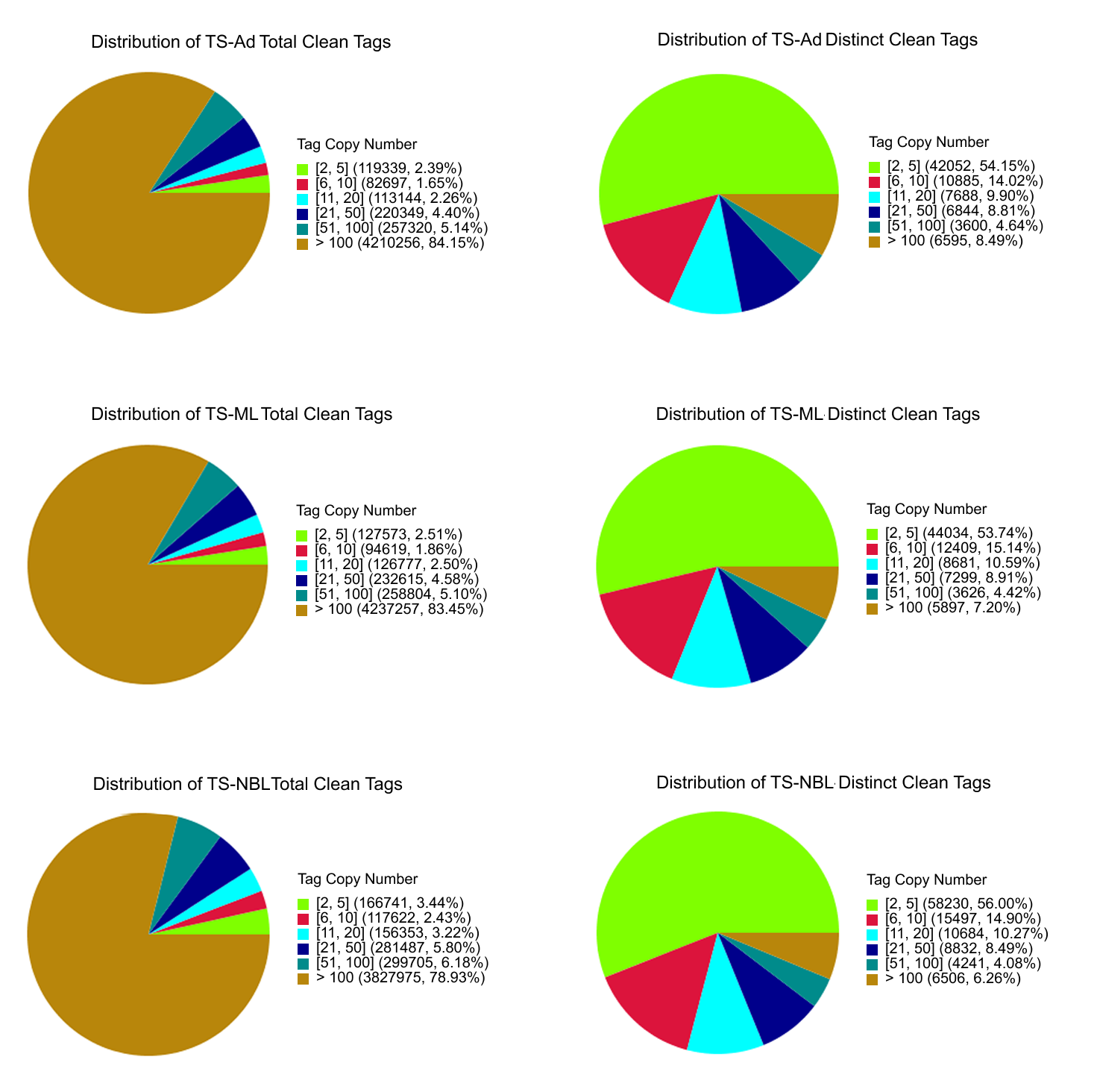

Supplement: File S2 — Distribution of total clean tags and distinct clean tags in each DGE library. The numbers in square brackets indicate the range of copy numbers for each category of tags. The data in parentheses indicate the number and percentage of corresponding tags among the total or distinct clean tags. Distribution of total clean tags and distinct clean tags in NBL, ML and Ad stages are presented. (TIF) [file pntd.0001794.s002.tif]
